# Radiotherapy for inverted papilloma: a case report and review of the literature

**DOI:** 10.2478/v10019-012-0045-8

**Published:** 2013-02-01

**Authors:** Primoz Strojan, Simona Jereb, Imre Borsos, Jasna But-Hadzic, Nina Zidar

**Affiliations:** 1 Department of Radiation Oncology, Institute of Oncology Ljubljana, Ljubljana, Slovenia; 2 Department of Radiology, Institute of Oncology Ljubljana, Ljubljana, Slovenia; 3 Clinic for Otorhinolaryngology and Cervicofacial Surgery, Clinical Center Ljubljana, Ljubljana, Slovenia; 4 Institute of Pathology, Medical Faculty University of Ljubljana, Ljubljana, Slovenia

**Keywords:** inverted papilloma, local control, radiotherapy, surgery

## Abstract

**Background:**

Sinonasal inverted papilloma (IP) is a rare, usually benign tumor arising from the respiratory mucosa of the sinonasal tract. Surgical resection is the treatment of choice. In histologically overt benign IPs (*i.e.* without associated malignancy) irradiation was employed only anecdotally. The patient with gross residual of benign IP after up-front surgery that was subsequently treated with irradiation is presented and the literature reports on the use of radiotherapy (RT) in this tumor type are reviewed.

**Case report:**

After the surgical treatment the residuum in the region of the sphenoid and adjacent cavernous sinus was irradiated to 54 Gy in 1.8 Gy daily fractions. No recurrence or deterioration of olfaction, hearing or vision was observed 2.6 years post-RT.

**Review of the literature:**

In the literature, six reports were identified with 16 patients describing necessary details on RT and outcome. Twelve of 14 cases (our case included) with gross or subtotal tumor resection and postoperative RT were locally controlled. The lowest and the median irradiation doses were 47.15 Gy and 56.5 Gy, respectively, and the follow-up period ranged between 0.5–20.5 years (median 7.8 years).

**Conclusions:**

RT is safe and valuable treatment option in histologically overt benign IPs. It is indicated when the risk of tumor recurrence after surgery is increased and in inoperable tumors.

## Introduction

Sinonasal inverted papilloma (IP) is by far the most frequent histologic type of Schneiderian papillomas, a group comprising also of fungiform and oncocytic variants, which altogether represent 0.4–4.7% of all sinonasal tumors.^1^ From an etiological perspective, the formation of IPs have been linked to human papillomavirus (HPV) infection, although there are other opinions suggesting that HPV more likely represents incidental colonization than being an important etiological factor.^2^,^3^ Associated malignancy, most often of squamous cell histology, may arise from IPs or may appear concomitantly with IPs in 11% of cases.^1^

A typical patient with IP is male in his fifth to seventh decade of life presenting with a unilateral nasal obstruction. Clinical examination usually reveals an unilateral polypoid tumor that originates from the lateral nasal wall in the region of the middle turbinate or ethmoid recesses although extensive lesions destroying neighboring structures could also be seen at the initial presentation.^1^ Surgical resection is the treatment of choice for benign IPs. Endoscopic treatment is preferred if the attachment site, preoperatively identified by the CT finding of focal hyperostosis, could be confirmed intraoperatively and adequately reached by the instrument.^4^,^5^

For tumors with associated malignancy such as IP with squamous cell carcinoma (SCC) there is a generally accepted view that RT adjuvant to surgical resection contributes to better local control.^6^ In benign IPs, however, irradiation has been employed only anecdotally. In this report, we present our experience with RT for gross residual benign IP after up-front surgery and a summarization of literature reports on the use of RT in this tumor type.

## Case report

A 69-year old male, heavy smoker and abstainer, was presented in January 2009 with a 5-year history of progressive nasal obstruction, anosmia lasting one year and diplopia lasting two weeks. Thirty years prior to referral he suffered from an acute myocardial infarction with a cardiac by-pass inserted 5 years ago; since that time he has been under the regular surveillance of a cardiologist.

Upon clinical examination, extensive polyps occluding the middle nasal meatus bilaterally were seen. In addition, double vision was recorded when the patient looked to the left and inferiorly along with a left eye small visual field defect temporally. Rinne’s test was positive on the right and negative on the left ear with lateralization to the left upon Weber testing. The remainder of the physical examination and the results of laboratory tests were unremarkable.

**FIGURE 1.**
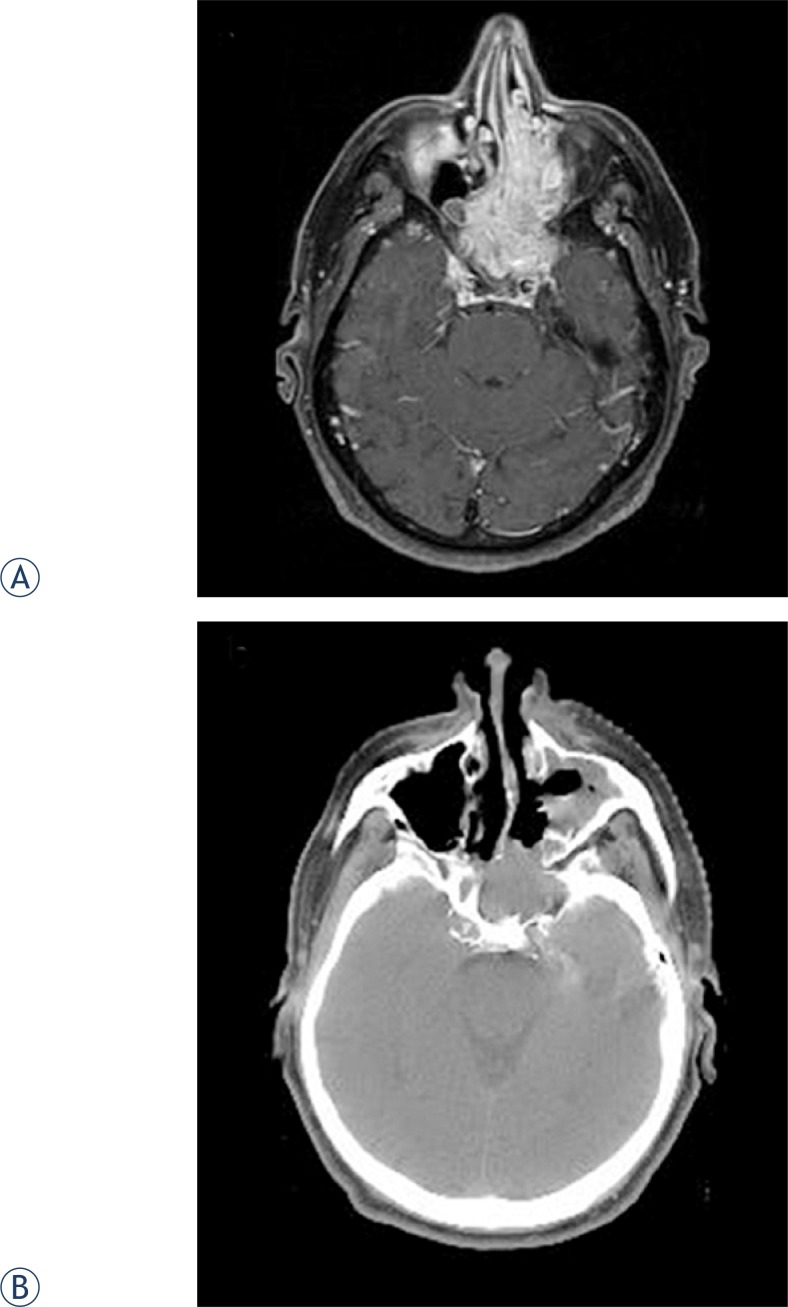
A. Before therapy (post-contrast MRI T1 SE WI with fat suppression). B. After subtotal resection of tumor (native CT scan).

**FIGURE 2.**
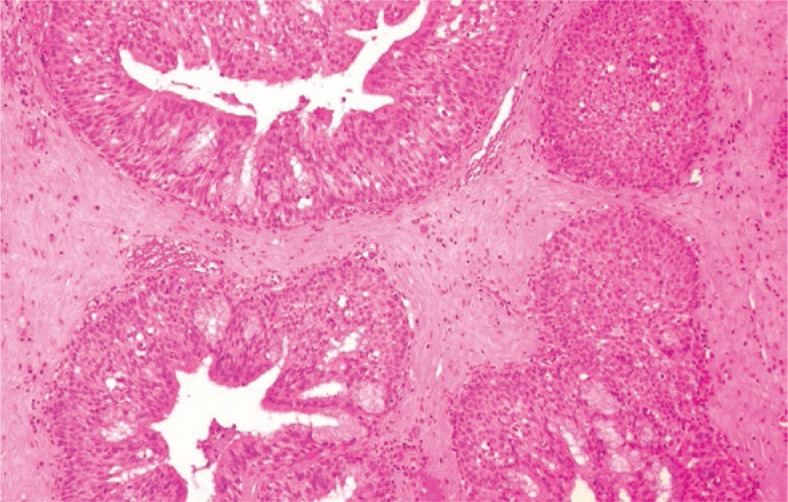
Inverted papilloma: invagination of nonkeratinizing squamous and pseudostratified columnar ciliated epithelial cells into the subepithelial stroma (H&E, orig. magnification × 100).

Contrast enhanced magnetic resonance imaging (MRI) revealed a 7.5x4.5x5.5 cm tumor mass involving the sphenoid sinuses, nasal cavity and ethmoidal cells bilaterally. Cranially the tumor extended to the optic chiasm pushing apart optic nerves; it infiltrated the cavernous sinus on the left and the clivus posteriorly but no dural or brain invasion were documented. The tumor was also present in the posterior part of the left maxillary sinus and the nasopharynx (Figure 1A). A biopsy was taken and a histological examination was consistent with the diagnosis of IP with no dysplastic or malignant changes present (Figure 2). In situ hybridization for HPV 16/18 and 6/11 was negative.

The patient underwent Caldwell-Luc surgery on the left side with tumor being removed from both nasal cavities, the nasopharynx and left maxillary, ethmoid and part of the sphenoid sinuses. Due to the vicinity of the pituitary gland and infiltration of the left cavernous sinus, no attempt was made to resect intracranial part of the tumor. A residuum of 3.5 x 3 cm was left behind in the region of the sphenoid and adjacent cavernous sinus with minimal extension into the posterior part of the nasal cavity as was documented on a postoperative computer tomography (CT) scan (Figure 1B). At the same time, revision of the left frontal sinus did not reveal a visible tumor which was confirmed with biopsy specimens being negative for IP. Postoperatively, the patient was irradiated using CT-based 3-dimensional computer planning and five 6 MV linear accelerator photon beams. The clinical target volume encompassed the residual tumor and a margin of 3 mm was added to create a planning target volume. A tumor dose of 54 Gy was delivered in 30 daily fractions of 1.8 Gy over 45 days.

**FIGURE 3.**
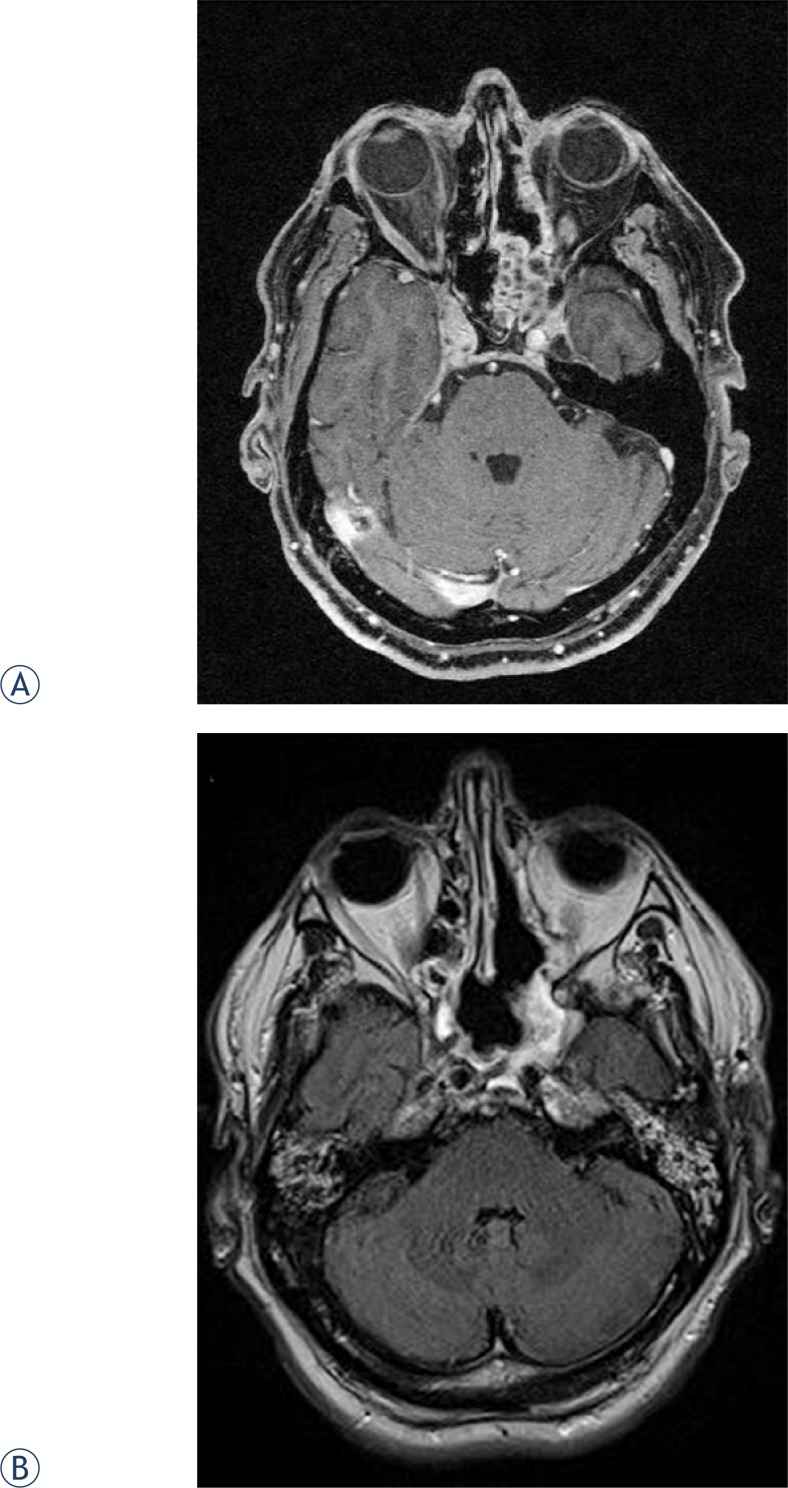
A. Six months after radiotherapy (post-contrast MRI T1 GE WI with fat suppression). B. Three years after diagnosis (post-contrast MRI T1 SE WI with fat suppression).

Three months after finishing irradiation, a control MRI did not demonstrate any significant change in tumor size when compared to postoperative CT. Three months later, however, a substantial reduction in tumor mass was observed on an MRI with a tumor residue of 3 × 2 cm encompassing the sphenoid and left cavernous sinus (Figure 3A) that remained unchanged on a control MRI 9 months post-therapy. Additional shrinkage of the tumor was documented at radiologic controls one and two years later with residual changes in the area of the left sphenoid sinus appearing more homogenous when compared to previous scans (Figure 3B). No deterioration of olfaction, hearing or vision was observed in the patient at the last follow up examination in January 2012.

## Review of the literature

A systematic review of the English literature was accomplished by using the PubMed database and the search terms *inverted papilloma, radiotherapy* and *irradiation.* In the publications displayed, content was reviewed for possible inclusion and references were checked for additional relevant reports. The criteria for inclusion of the article in the present review were availability of data on RT procedures, survival times and status at the last follow-up of reported IP patients.

Altogether, six reports were identified with 16 patients that met the inclusion criteria (Table 1).^7^–^12^ According to the volume of tumor that was irradiated, patients (including our case) were grouped as follows: (*i*) no residual tumor visible after gross tumor resection – 9 cases; (*ii*) macroscopic residual tumor after subtotal resection – 5 cases; and (*iii*) macroscopic tumor with no surgery performed – 3 cases. RT doses in the first two groups ranged from 50 Gy to70 Gy (median 56.6 Gy) and from 47.15 to 68 Gy (median 56.4 Gy), respectively, whereas in three patients who only had a biopsy prior to irradiation, the doses were 50 Gy, 65 Gy and 68 Gy. Local recurrence occurred in four cases, one from each group with surgery, (after 50 Gy, 57.4 Gy) and in two cases that were only irradiated (after 50 Gy and 65 Gy). Salvage surgery was successful in two patients^7^,^8^ and associated malignancy was documented subsequently during the course of the disease in another patient.^11^

## Discussion

Radiotherapy has only been used on exception in the treatment of histologically overt benign IPs. One reason is historical: in 1965, Mabery *et al*. identified in the literature four out of 14 patients with a malignant transformation in papillomatosis who had a history of RT.^13^ Sporadic cases of assumed RT-induced malignancy in previously benign IPs were also reported by others.^14^–^16^ Due to the risk of anaplastic transformation after treatment with irradiation, many authors in the past advocated avoiding the use of RT in this type of tumor. However, this argument should be considered non-relevant in the context of more recent data as no such relationship could be confirmed in the majority of IP/SCC cases.^8^,^10^,^17^

The second argument, also originated in the past when imaging and RT were much less sophisticated and effective than nowadays, would be the opinion that RT is ineffective in preventing recurrences.^4^,^18^,^19^ Favorable results, however, were also described with RT by others.^8^–^10^,^12^,^17^,^20^ Lastly, high local control rates achieved by surgery eliminate the need for adjuvant therapy.^5^,^21^ Recently, modern radiologic techniques (*e.g*. CT, MRI) allow even better visualization of the extent of tumors and more effective planning of surgical procedures.

In reviewing the literature for reports on irradiation of benign IPs, we found only six studies with, in total, 16 patients describing necessary details on therapy and outcome (Table 1).^7^–^12^ Indications for irradiation were inoperability, due to the extent of a tumor or medical comorbidities, incomplete resection or history of multiple recurrences of otherwise benign tumor. When only those patients, after gross or subtotal tumor resection from Table 1 are considered, RT failed in 2 out of 14 cases (our case included). The two were irradiated to 57.4 Gy and 50 Gy.^8^,^11^ The first patient was treated in 1970s, when imaging and RT technologically inferior to the present standards were employed.^8^ In the latter case, however, a malignant component was identified 12 months later when the patient was re-operated on due to a subsequent recurrence. This may provoke speculation on preexisting but originally overlooked malignant features in the tumor that require doses well above 50 Gy.^6^ In patients who were locally controlled, the lowest and the median dose were 47.15 Gy (gross residual, split-course RT) and 56.5 Gy, respectively, and the follow-up period ranged between 0.5–20.5 years (median 7.8 years). It seems that in overt benign IPs RT with doses ranging from 50 Gy to well below 60 Gy effectively prevent tumor re-appearance when there is no residual or only a small residual left behind after surgery. If tumor burden is extensive, *e.g*. when no surgical de-bulking is carried out, irradiation doses in the range of 70 Gy appear to be indicated.

In the presented patient, infiltration of the cavernous sinus prevented gross resection of the tumor and subsequent RT resulted in persistent local control of 2.6 years post-therapy. Importantly, the RT dose that was used (54 Gy in 30 fractions of 1.8 Gy per day) is still in the range of doses closer to a “near zero” incidence of RT-induced optic neuropathy,^22^ which makes RT acceptable also from a morbidity point of view. Hyperfractionation of RT dose and modern RT techniques with increased dose conformity (*e.g*. intensity-modulated RT with image guidance) can further reduce the risk of optic neuropathy.^23^,^24^ Because only two cases with neck metastases of benign-appearing IPs have been reported so far^7^,^25^, no elective irradiation of regional lymphatics is needed.

In our patient, a reduction in tumor mass was seen on an MRI scan no earlier than six months post-RT, and this trend continued at radiologic controls one and two years after treatment. This observation points to the need for a sufficient post-RT interval before it is found to be ineffective.^10^ To date, no late recurrences have been described among IP patients after irradiation although this is obviously not the case in the IP/SCC group.^6^,^10^,^17^

To conclude, in histologically overt benign IPs, RT is safe and is indicated when the risk of tumor recurrence after surgery is increased, either due to subtotal resection or a history of recurrent disease, and in inoperable tumors. Moderate RT doses well below 60 Gy can effectively prevent recurrence after gross or subtotal resection whereas for inoperable tumors doses in the range of 70 Gy are indicated. Tumor response after irradiation should be assessed not earlier than 3–6 months after treatment.

## Figures and Tables

**TABLE 1.** Radiotherapy for sinonasal inverted papilloma: review of the literature

**Author, (year)[Ref]**	**Sex/Age (years)**	**Prior surgery**	**Tumor extent**	**Surgery**	**Radiotherapy**	**Post-radiotherapy status**
Fechner & Sessions (1977)^7^	F/22	No	Rt medial canthal area & Rt neck mass	Primary tumor – no & excision of neck mass	50 Gy	PD locally at 1.8 years →NED, 3 years (after SURG)
Hug *et al.* (1993)^8^	7 patients, M (all 7)/30–70	Yes, ≤8 (in 5 patients) No (in 2 patients)	ES 72%, MS 68%, SS 36%, FS 20%, nasopharynx 16%; Neighboring structures^a^ 52%	Gross total resection, 3 patients	51.2 Gy, bid 54.6 Gy, qd 56.6 Gy, qd	NED, 6.3 years NED, 7.2 years NED, 8.4 years
				Subtotal resection, 3 patients	56.4 Gy, bid 57.4 Gy, bid 64.8 Gy, qd	NED, 0.5 years PD→NED, 5.2 years (after repeated SURG) NED, 12.9 years
				No, 1 patient	68.4 Gy, qd	NED, 3.5 years
Miller *et al.* (1996)^9^	F/42	Yes, 1	Lt-FS, IC, dura	Gross total resection	70 Gy	NED, 3 years
Gomez *et al.* (2000)^10^	M/40	Yes, 3	Lt-NC, Lt-ES, Lt-SS, cribriform plate, Lt&Rt-FS, IC	Subtotal resection, gross residual tumor	47.15 Gy, 32 fx, qd (sc, over 78 day)	DOC (lung carcinoma), 20.5 years
	M/56	Yes, 2	Lt-MS, ES, cribriform plate	Gross total resectio equivocal margins	67 Gy, 60 fx, bid	NED, 9 years
	M/47	No	Lt-NC, Lt-MS, ES	Gross total resection microscopic residual	61.3 Gy, 32 fx, qd (sc, over 58 days)	DOC (lung carcinoma), 9 years
	F/32	Yes, 1	Lt-ES, Lt-MS, cribriform plate, medial orbital wall	Gross total resection	60 Gy, 33 fx, qd	NED, 8.5 years
	F/84	Yes, 1	Lt-NC, Lt-MS, Lt-ES, Lt-orbit, Lt-FS	No	65 Gy, 36 fx, qd	DOD, 1.4 years
Acevedo-Henao *et al.* (2010)^11^	M/63	Yes, 2	Rt-MS, Rt-middle ear, temporal fossa, n.VII	Gross total resection	50 Gy, 25 fx, qd	DOD^b^, 2.2 years
Kainuma *et al.* (2011)^12^	M/63	Yes, 4	Lt-middle ear	Gross total resection	54 Gy	NED, 0.8 years
Present report	M/69	No	NC, EC, SS, Lt-MS, Lt-cavernous sinus	Subtotal resection, gross residual tumor	54 Gy, 30 fx, qd	NED, 2.6 years

M = Males; F = Females; ES = Ethmoid sinus; MS = Maxillary sinus; SS = Sphenoid sinus; FS = Frontal sinus, IC = Intracranial; NC = Nasal cavity; n.VII = Facial nerve; Lt = Left; Rt = Right; bid = twice-a-day irradiation; qd, = once-a-day irradiation; fx = fraction; sc = split-course

a Neighbouring structures: orbit, cribriform plate, infratemporal fossa, clivus, pterygomaxillary space, palate, or cheek.

b At the time of RT, histologic diagnosis was benign IP; subsequently, associated squamous cell carcinoma was found during the course of the disease
